# Antioxidant Capacity of Two Novel Bioactive Fe(III)-Cyclophane Complexes

**DOI:** 10.3390/molecules18021762

**Published:** 2013-01-29

**Authors:** Alex J. Salazar-Medina, Rocío Sugich-Miranda, Eli Teran-Cabanillas, Jesús Hernández, Gustavo A. González-Aguilar, Enrique Rudiño-Piñera, Rogerio R. Sotelo-Mundo, Enrique F. Velázquez-Contreras

**Affiliations:** 1 Departamento de Investigación en Polímeros y Materiales (DIPM), Universidad de Sonora, Calle Rosales y Blvd, Luis Encinas s/n, Col. Centro, PO Box 130. Hermosillo Sonora 83000, Mexico; 2 Centro de Investigación en Alimentación y Desarrollo (CIAD), A.C. Carretera a Ejido La Victoria Km 0.6, PO Box 1735, Hermosillo Sonora 83304, Mexico; 3 Departamento de Medicina Molecular y Bioprocesos, Instituto de Biotecnología, Universidad Nacional Autónoma de México (IBT-UNAM) Av. Universidad #2001, Col. Chamilpa, Cuernavaca, Morelos 62250, Mexico

**Keywords:** cyclophane, macrocycle, iron, biomimetic, antioxidant

## Abstract

The cyclophanes 2,9,25,32-tetraoxo-4,7,27,30-tetrakis(carboxymethyl)-1,4,7,10,24,27,30,33-octaaza-17,40-dioxa[10.1.10.1]paracyclophane (**PO**) and 2,9,25,32-tetraoxo-4,7,27,30-tetrakis(carboxymethyl)-1,4,7,10,24,27,30,33-octaaza[10.1.10.1]paracyclophane (**PC**) were coordinated with iron to form cationic binuclear Fe(III) ****Fe_2_PO**** and ****Fe_2_PC**** complexes, respectively. Their antioxidant capacity, superoxide dismutase and peroxidase mimetic activity, as well as their toxicity toward peripheral blood mononuclear cells (PBMCs) were evaluated. Both ****Fe_2_PO**** and ****Fe_2_PC**** are interesting biomimetics with antioxidant capacity similar to that of ascorbic acid that prevent mortality in cultured PBMCs, with the potential to have bioactive and protective functions in disease animal models.

## 1. Introduction

Reactive oxygen species (ROS) are toxic molecules implicated in several human pathological processes including tissue injury, inflammation, ageing, cancer, cardiovascular, pulmonary and neurodegenerative diseases [1]. Aside from many enzymatic and chemical antioxidants, several synthetic metal complexes have shown activity as biomimetic antioxidants [2]. Transition metal complexes [e.g., complexes of Mn(II), Mn(III), Cu(II) and Fe(III)] have important antioxidant properties [1], due to their ability to change their oxidation state and their chemical interactions with a wide number of negatively charged ions, radicals and molecules. These characteristics have prompted the design and development of metal-based drugs with promising pharmacological application which might offer unique therapeutic opportunities [3].

The first line of defense against ROS in cellular systems is superoxide dismutase (SOD), that catalyzes the conversion of O_2_^•−^ to H_2_O_2_ and O_2_ [4]. Some macrocyclic complexes have been proposed as SOD mimetics, mainly because of their high thermal stability and flexible ligand arrangement that allows redox processes based on their metal centers [5]. The action of SOD produces H_2_O_2_ by the O_2_^•−^ dismutation, a much less reactive molecule compared to O_2_^•−^, but still toxic to the cell. H_2_O_2_ in the presence of transition metals, it is rapidly converted via the Fenton reaction to the hydroxyl radical (^•^OH). The ^•^OH is widely accepted to be the most damaging and cytotoxic ROS produced by cells. Two enzymes participate in the removal of H_2_O_2_ from the cellular environment, peroxidases (POx) and catalases (CAT), converting the H_2_O_2_ into water molecules and diatomic oxygen [6]. For these reasons we examined the use of Fe(III) ions coordinated to cyclophane ligands generating a new binuclear Fe(III)-cyclophane group of complexes, which could represent a new class of antioxidants. The study of these molecules will provide a deeper insight into the understanding of complexes that could mimic some enzymes related to ROS contention mechanisms.

## 2. Results and Discussion

### 2.1. Synthesis of the PO and PC Iron Complexes

**Figure 1:**
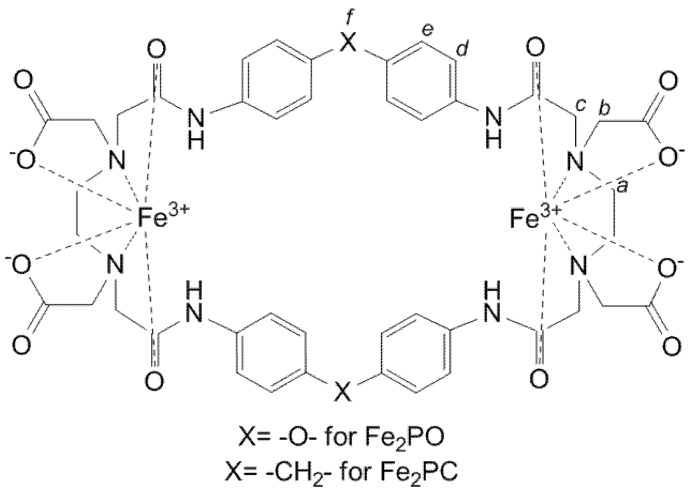
Chemical structures proposed of ****Fe_2_PO**** and ****Fe_2_PC**** cyclophane iron (III) complexes.

The purified chelating cyclophanes 2,9,25,32-tetraoxo-4,7,27,30-tetrakis (carboxymethyl)-1,4,7,10,24,27,30,33-octaaza-17,40-dioxa[10.1.10.1] paracyclophane (**PO**) and 2,9,25,32-tetraoxo-4,7,27,30-tetrakis(carboxymethyl)-1,4,7,10,24,27,30,33-octaaza[10.1.10.1] paracyclophane (**PC**), were synthesized as previously reported [7]. Both molecules were derived from ethylenediaminetetraacetic acid (EDTA) and provided with amino, amide and carboxymethyl pendant donor groups. Their purity was confirmed by melting/decomposition point (MDP) and NMR studies (see Experimental section below). Reactions between FeCl_3_.6H_2_O (at pH ~ 3) and **PO** and **PC** (both at pH ≤ 7), gave binuclear Fe(III) complexes [Fe_2_L]^2+^, labeled ****Fe_2_PO**** and ****Fe_2_PC****, respectively (Figure 1). We propose that **PO** and **PC** metal chelates have a structure in which each Fe atom is coordinated by two carboxylate oxygens, two amine nitrogens and two amide oxygen atoms, with a feasible water molecule axial coordinated in aqueous solutions, on the basis of the well-known ferric EDTA complexes [8] and the coordination mode found in the X-ray crystal structure of a related cyclophane (3,10,21,28-tetraoxo-5,8,23,26-tetrakis(carboxymethyl)-2,5,8,11,20,23,26,29-octaaza[12.12] paracyclophane) bound to zinc [9]. ****Fe_2_PO**** was soluble in aqueous solutions in a pH range from 4.2 to 10.0, while ****Fe_2_PC**** was soluble in aqueous solutions in a pH range from 5.2 to 10.0.

### 2.2. Mass Spectrometry

To confirm the formation of the metal complexes, the mass spectra of methanol-water solutions of **Fe_2_PO** and **Fe_2_PC** were obtained. **Fe_2_PO** showed a major peak assigned to [Fe_2_^3+^L^4−^]^2+^ specie at *m/z* = 510 with an abundance of 100%. Another peak that supports the formation of the binuclear Fe(III) complex correspond to [Fe_2_^3+^L^4−^Cl^2−^] at *m/z* = 1,092 with an abundance of 6%. For **Fe_2_PC** a major peak assigned to [Fe_2_^3+^L^4−^]^2+^ specie was observed at *m/z* = 508 with an abundance of 100%. Molecular ion peaks corresponding to mononuclear species [Fe^3+^L^−4^]^−^ were not detected for **Fe_2_PO** or **Fe_2_PC**.

### 2.3. Electron Paramagnetic Resonance (EPR)

**Figure 2:**
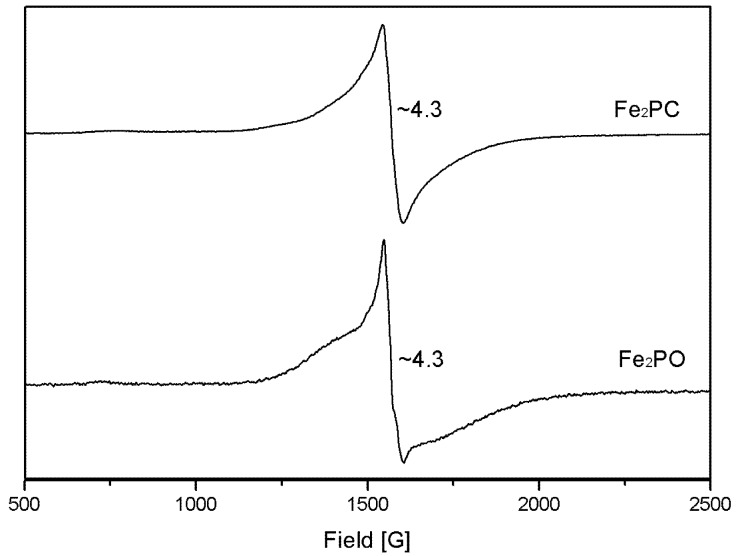
g values of the X-band EPR spectra for **Fe_2_PO** and **Fe_2_PC**. Experimental conditions: methanol/water (6:4) matrix, temperature 6 K, microwave frequency 9.338 GHz, modulation 1 G.

The X-band EPR spectra observed for **Fe_2_PO** and **Fe_2_PC** in 60% aqueous methanol solutions at 6 K are shown in Figure 2. The spectra exhibit an intense peak at g ~ 4.3 in the region of 1,500 G, typical of high-spin (*S* = 5/2) iron(III) complexes with low symmetry, as well as commonly found in a variety of solid state materials, chelates and metallo proteins [10,11].

### 2.4. Infrared Spectroscopy

**Figure 3:**
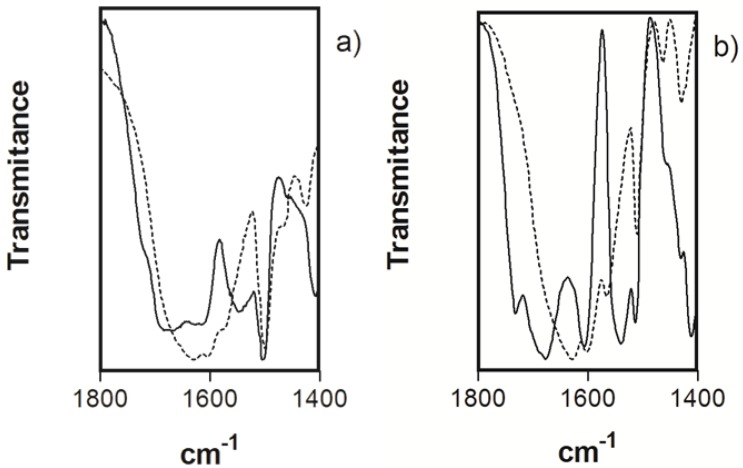
IR spectra for KBr pellets of (**a**) **PO** and **Fe_2_PO** (solid and dotted line respectively); and (**b**) **PC** and **Fe_2_PC** (solid and dotted line respectively).

The coordination mode for **Fe_2_PO** and **Fe_2_PC** complexes was analyzed by IR spectroscopy by comparison of absorption bands of the non-coordinated ligand *vs*. the metal complex (Figure 3). The stretching carbonyl bands gave shifts from 1,684 cm^−1^ (**PO**) to 1,620 cm^−1^ (**Fe_2_PO**) and 1,674 cm^−1^ (**PC**) to 1,624 cm^−1^ (**Fe_2_PC**). We also observed a broad OH band at the 3,200–3,600 cm^−1^ region. The changes in the position of the absorption bands of Fe-bound and unbound cyclophanes provide us an indicative of the formation of the metal complex and its preferred coordination sites in the ligand structures, as well as the observed for Fe(III)-EDTA complexes [8].

### 2.5. Electronic Absorption Spectra

**Figure 4:**
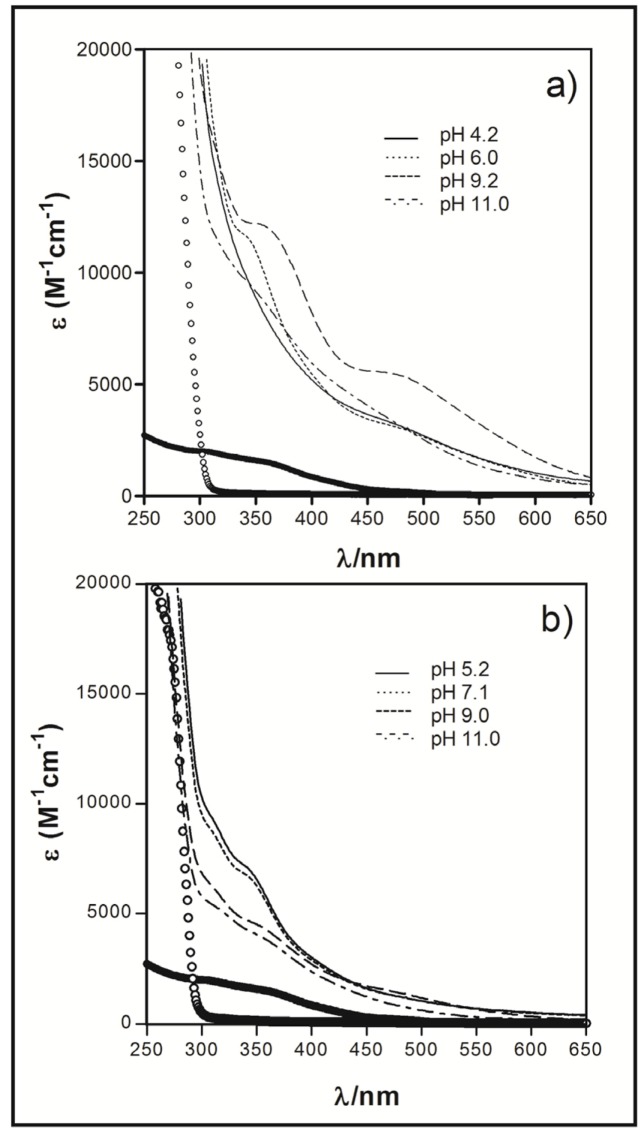
Solution electronic spectra of (**a**) **Fe_2_PO** and (**b**) **Fe_2_PC** at different pH values. White and black circles spectra corresponded to unbound cyclophane and Fe(III) respectively, both at pH ~ 7, as a controls.

**Figure 5:**
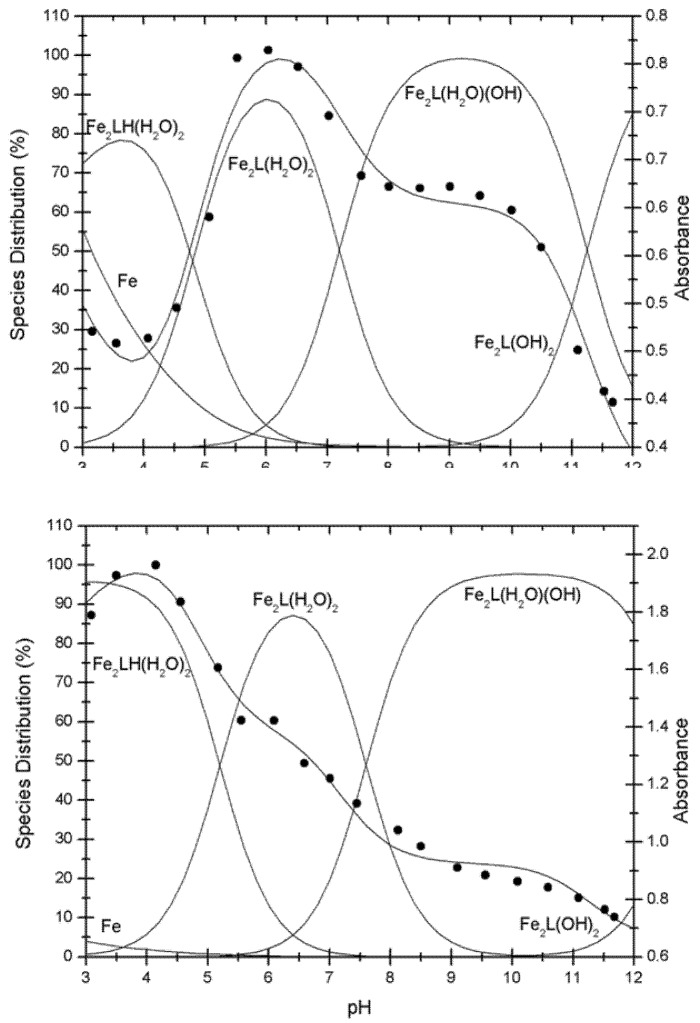
Correlation between the species distribution and the absorption spectra of **Fe_2_PO** at top and **Fe_2_PC** at bottom. (% = Percentage of species distribution in solution).

The electronic spectra for **Fe_2_PO** solution were pH dependent, as shown in Figure 4(a). In a pH range of 6.0 to 10.0 the spectra show two bands with a λ_max_ of 350 nm (ε_pH7.0_ = 11,830 M^−1^cm^−1^) and 475 nm (ε_pH7.0_ = 4,680 M^−1^cm^−1^), assigned to a ligand-to-metal charge transferences (LMCT) [12]. With an increase of the pH, the molar absorptivity also increases, and the λ_max_ of each band was slightly displaced to a major wavelengths. Above pH ~ 10, the molar absorptivity of the LMCT bands decreases due to the precipitation of the complex in the working solution. The data obtained in the UV-Vis experiment were analyzed through the program Excel^®^ Worksheets for Spectrometry [13] and we determined the presence of different protonation state species of the **Fe_2_PO** complex [Figure 5(a)], where the non-deprotonated species Fe_2_L(H_2_O)_2_ reaches its maximum concentration at pH 6.0 and then start to decrease. Over this pH, the **Fe_2_PO** complex solution becomes into a dark red colored solution and the mono deprotonated species Fe_2_L(H_2_O)(OH) and bi deprotonated species Fe_2_L(OH)_2_ appears, to finally shift into a yellow colored suspension (precipitate complex) at pH 11.0. These spectrophotometric observations are consistent with those corresponding to the complex Fe(III)-EDTA [14].

The electronic spectra of **Fe_2_PC** also present two LMCT bands, the first one at 307 nm (ε_pH7.0_ = 8,710 M^−1^cm^−1^), and the second band at 334 nm (ε_pH7.0_ = 6,270 M^−1^cm^−1^). In this case, the intensity of the two bands decreases with the augmentation of the pH [Figure 4(b)]. The same method of analysis was used to establish the presence of different protonation state species of **Fe_2_PO**, and it was utilized with the UV-Vis data spectra of **Fe_2_PO** complex [Figure 5(b)]. The non-deprotonated species Fe_2_L(H_2_O)_2_ moves its maximum concentration percentage from pH 6.0 for **Fe_2_PO** to pH 6.5 for **Fe_2_PC** and then starts to decrease. Over pH ≈ 8.0 the deprotonated species appears, Fe_2_L(H_2_O)(OH) and Fe_2_L(OH)_2_ , and the complex solution becomes a dark red colored solution, to finally shift into a yellow colored suspension due to the formation of the precipitate complex over pH 11.0, as well as that observed to **Fe_2_PO**.

**Figure 6:**
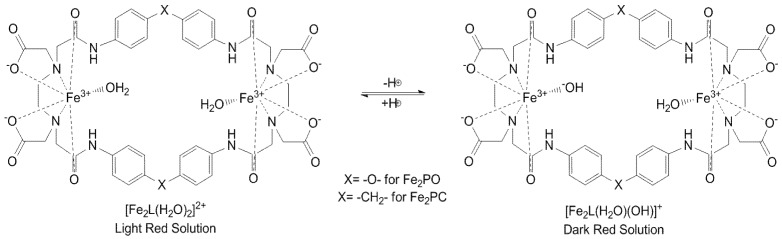
Changes in protonation state of the aqueous solutions of **Fe_2_PO** and **Fe_2_PC** complexes in dependence of pH.

As a consequence of the pH dependence mentioned above, a mixture of the species Fe_2_L(H_2_O)_2_ and Fe_2_L(H_2_O)(OH) of **Fe_2_PO** as well as **Fe_2_PC**, was present in each experiment described below (Figure 6).

The differences between the substituent groups X = -O- for **Fe_2_PO** and X = -CH_2_- for **Fe_2_PC** (Figure 1), provide the Fe(III) complexes with distinct properties, even with the no participation of the substituent groups in the coordination sphere with iron. X = -O- allows a major flexibility to **Fe_2_PO** against the **Fe_2_PC**, because the more rigidity property of X = -CH_2_- and this characteristic is responsible of the differences in the UV-Vis spectra behavior between **Fe_2_PO** and **Fe_2_PC**. Even though the two iron atoms coordinate to the same groups in **PO** and **PC** ligands, a higher tension was expected to **Fe_2_PC** complex to maintain the coordination. In addition, **Fe_2_PO** has shown major solubility in aqueous solution, letting us to study it at lower pHs than Fe_2_PC do. In the species distribution diagram we can see the non-protonated and protonates species of **Fe_2_PO** and **Fe_2_PC** appear at different pH values, behavior attributable to the structural rigidity in each metal complex.

### 2.6. Antioxidant Capacity Assay

**Figure 7:**
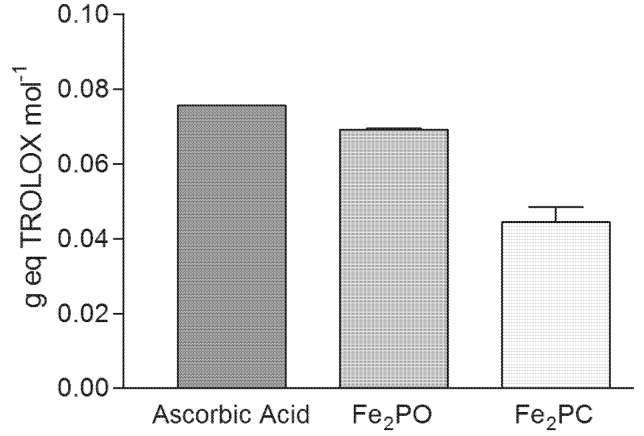
Antioxidant capacity of **Fe_2_PO** and **Fe_2_PC** complexes by the TEAC assay. Ascorbic acid and a solution of Fe(III) were used as a controls. Bars represent the standard error of the mean (±SEM) of triplicate experiments. Ferric chloride had no detectable antioxidant capacity and was not plotted.

The characterization of antioxidant capacity is of great importance because the multiple biological and medical applications of such synthetic molecules. The **Fe_2_PO** had more antioxidant capacity (0.0690 g eq Trolox mol^−1^) than **Fe_2_PC** (0.0443 g eq Trolox mol^−1^), both within the range of the antioxidant capacity presented by ascorbic acid at the same molar concentration (0.0755 g eq Trolox mol^−1^) (Figure 7). The behavior of the Fe(III) complexes was similar to the observed for the Cu(II) complexes reported by Sugich-Miranda and co-workers [5]. The difference between **Fe_2_PO** and **Fe_2_PC** antioxidant capacity could be due to differences in conformational flexibility of **Fe_2_PC**, resulting in a less favorable interaction with the ABTS^•+^ radicals in solution causing a lower antioxidant capacity. The antioxidant capacity of iron (FeCl_3_) was undetectable in the TEAC assay.

### 2.7. Superoxide Dismutase-Like Activity

**Figure 8:**
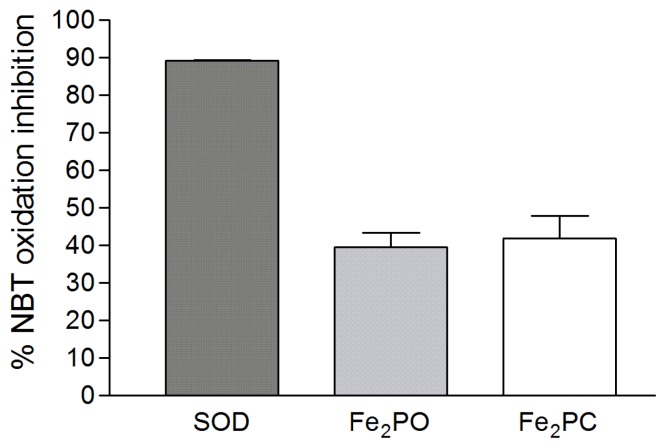
SOD-like activities of **Fe_2_PO** and **Fe_2_PC** complexes as inhibitors of the NBT oxidation. Bars represent the standard error of the mean (±SEM) of triplicate values.

The SOD-like activity was measured by inhibition of the oxidation of nitrobluetetrazolium (NBT) induced by the presence of the O_2_^•−^ radical generated by the xanthine and xanthine oxidase enzymatic system. We tested bovine SOD as the main scavenger of O_2_^•−^ radical, producing 89.40% of NBT oxidation inhibition with a 60 µM concentration of the enzyme. Under the same conditions the NBT oxidation inhibition of **Fe_2_PO** was 39.53% and for **Fe_2_PC** was 41.97% (Figure 8). The values obtained of both metal complexes, reaches over a 44% of NBT oxidation inhibition generated by bovine SOD.

### 2.8. Peroxidase-Like Activity

**Figure 9:**
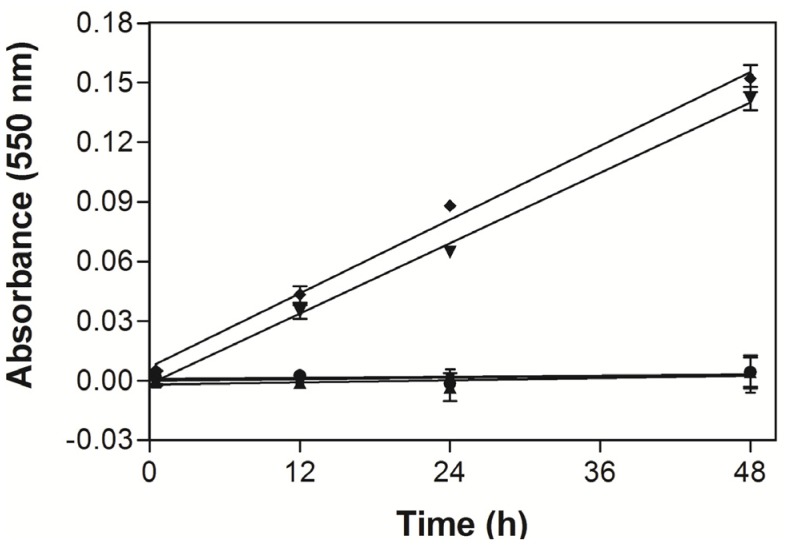
Peroxidase-like activity of ● FeCl_3_, ■ PO, _▲_ PC, _▼_ Fe_2_PO and ♦ Fe_2_PC. Bars represent the standard error of the mean (±SEM) of triplicate values.

In order to investigate the bioactive capacity as a POx mimetic, the POx-like activity of **Fe_2_PO** and **Fe_2_PC** complexes was determined using the Amplex^®^ Red Hydrogen Peroxide/Peroxidase Assay Kit. The results observed were 0.13 and 0.29 nm of Amplex^®^ Red oxidized min^−1^ mg^−1^ complex for **Fe_2_PO** and **Fe_2_PC**, respectively. Both **Fe_2_PO** and **Fe_2_PC** complexes kept the POx-like activity for about 48 h, as observed with a continuous oxidation of the Amplex^®^ Red reagent in presence of H_2_O_2_ (Figure 9). This prolonged capacity of **Fe_2_PO** and Fe_2_PC to act as a POx mimetic towards H_2_O_2_ is desirable in a chemical with pharmacological properties. For example, the HRPO, as the main peroxidase tested in this assay, was only active *in vitro* for 30 min, compared to the 48 h of the cyclophane biomimetic complexes. Likewise, for a hemoglobin recombinant protein of the plant *Arabidopsis thaliana*, another class of POx mimetic, a rapid inactivation was observed after 30 s, although the great initial specific activity [15]. Therefore, the study of the possible mechanisms of these mimetic chemicals will give useful knowledge for the medical and pharmacological area.

### 2.9. Cellular Assay

**Figure 10:**
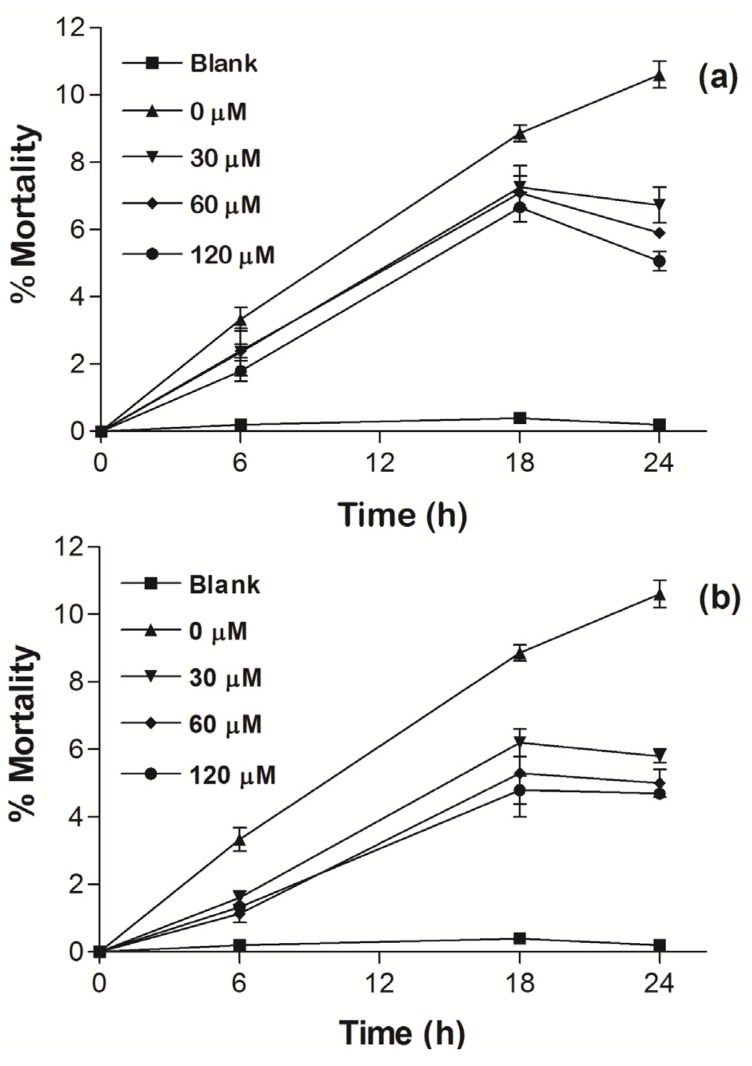
Cellular protection of (**a**) **Fe_2_PO** and (**b**) **Fe_2_PC** in PBMCs with induced damage by ROS. The line marked as blank corresponds to PBMCs in RPMI medium with no treatment. Data comes from a representative experiment. Points represent the standard error of the mean (±SEM) of triplicate values.

To evaluate the bio-protective effect of Fe(III)-cyclophane complexes, different concentrations of **Fe_2_PO** and **Fe_2_PC** (30, 60 and 120 µM) were incubated with PBMCs in the presence of xanthine plus xanthine oxidase to generate reduced species of oxygen and induce cell damage. The percentage of mortality was evaluated by trypan blue dye exclusion assay. Figure 10(a) shows that **Fe_2_PO** reduced mortality percentage in all the concentrations probed from 10.6% in control untreated cells to 5.06% with the highest concentration (120 µM) tested, after 24 h of culture. A similar trend was for **Fe_2_PC** [Figure 10(b)], reducing the mortality from 10.7% in control untreated cells to 4.70% with the highest concentration tested, after 24 h of culture. We previously evaluated the toxicity after 24 h culture of the Fe(III)-cyclophane complexes and the highest percentages of mortality for **Fe_2_PO** and **Fe_2_PC** was 0.9% and 0.5%, respectively. These results are similar to those previously reported by our group [5], where we found no toxic but a protective effect of the **Cu_2_PO** and **Cu_2_PC** in PBMCs by reducing mortality in 6% compared to control untreated cells. These results indicate that both cyclophane-Fe(III) complexes were not cytotoxic during the first 24 h of culture, even when high doses were used. Therefore, these molecules could be considered as potential candidates for further antioxidant studies using animal models and cancer cell lines in order to probe its possible beneficial effects. Over 24 h of culture the cell mortality increases because lacking of nutrients in the culture medium.

## 3. Experimental

### 3.1. Syntheses of Cyclophanes and Iron Complexes

**PO** was synthesized by adding bis(4-aminophenyl) ether (Aldrich, 1.5 g) in dimethylformamide (DMF, 70 mL) dropwise to ethylenediamine tetraacetic (EDTA) dianhydride (Aldrich, 2.0 g) in DMF (200 mL) under a nitrogen atmosphere. The light yellow solution obtained was concentrated and mixed with water, and a pale brown solid was obtained. This crude cyclophane was converted to the lithium salt, and recrystallized twice from hot water. Acidification (pH ~ 3) of the purified lithium salt gave **PO** in the acid form as a colorless solid. Yield: 15%. MDP: 262.0 °C. The absence of impurities such as oligomers was confirmed by ^1^H-NMR (D_2_O/Na_2_CO_3_, pD = 9.5, 400 MHz, DSS): δ_H_ = 2.86 (8H, s, Hb), 3.29 (8H, s, Ha), 3.43 (8H, s, Hc), 6.85 (8H, d, He), 7.18 (8H, d, Hd).

**PC** was synthesized by essentially the same method as for PO by the use of the diamine bis (4-aminophenyl)methane (Aldrich). The crude product was converted to the lithium salt, which was recrystallized from 50% ethanol. When an aqueous solution of the purified lithium salt was acidified with diluted HCl to pH ~ 3, **PC** in the acid form was obtained as a colorless solid. The product was washed with water. Yield: 12%. MDP: 256.0 °C. ^1^H-NMR (D_2_O, pD = 10.4; 400 MHz; DSS): δ_H_ = 2.81 (8H, s, Hb); 3.28 (8H, s, Ha), 3.38 (8H, s, Hc), 3.78 (4H, s, Hf), 6.98 (8H, d, He), 7.12 (8H, d, Hd).

To obtain the iron (III) complexes, each host molecule (either **PO** or **PC**) was dissolved in water and solubilized by adding a minimum amount of solid Na_2_CO_3_. This solution was added to an aqueous solution containing a slight excess of FeCl_3_·6H_2_O, the binuclear iron complex precipitated in a quantitative yield. The melting decomposition point (MDP) was obtained in a Mel-Temp II equipment (Laboratory Devices). The electrospray ionization (ESI) mass spectra were obtained by the use of a JEOL HX 110A spectrometer for sample solutions of methanol-water mixture.

**Fe_2_PO**. MDP: 301.7 °C. MS (ESI): *m/z* (for (C_44_H_44_Fe_2_N_8_O_14_)^2+^) = 510 (100) ([Fe_2_^3+^L^4−^]^2+^);

**Fe_2_PC**. MDP: 283.8 °C. MS (ESI): *m/z* (for (C_46_H_48_Fe_2_N_8_O_12_)^2+^) = 508 (100) ([Fe_2_^3+^L^4−^]^2+^).

### 3.2. Spectroscopic Measurements

The EPR spectra of the binuclear Fe(III) complexes were obtained on an X-band continuous wave Elexsys E500 (Bruker) EPR spectrometer, for 60% aqueous methanol solutions. The final concentration of each complex was approximately 1 mM. Infrared spectra were recorded on a Perkin Elmer Spectrum GX FT-IR system, in the range 4,000–400 cm^−1^. The samples were prepared as KBr pellets. The solution electronic spectra were obtained by the use of an Agilent 8453A UV-VIS diode array spectrometer. The pH of the samples solutions was adjusted by adding the necessary amount of NaOH solution. The species distributions were determined using the data obtained in the UV-Vis analysis varying the pH, with the program Excel^®^ Worksheets for Spectrometry [13].

### 3.3. Antioxidant Capacity Assay

The Trolox equivalent antioxidant capacity (TEAC) assay is based on determination of Trolox-equivalents, which is a water-soluble analog of vitamin E. Trolox (6-hydroxy-2,5,7,8-tetramethylchroman-2carboxylic acid) was purchased from Aldrich (Milwaukee, WI, USA). ABTS (2,2-azino-bis(3-ethylbenzthiazoline-6-sulfonic acid), potassium persulfate (K_2_S_2_O_8_) and ascorbic acid (vitamin C) were obtained from Sigma Chemical Co. (St. Louis, MO, USA). All other reagents were analytical grade or better. **Fe_2_PO** and **Fe_2_PC** complexes were evaluated at 30 and 60 µM concentrations using a modified TEAC method to determine their antioxidant activity. The TEAC method measures the antioxidant capacity to scavenging the blue-green ABTS^•+^ radical cation versus the scavenging capacity of Trolox. The radical cation ABTS^•+^ was obtained by mixing 5 mL of 7 mM ABTS solution and 88 µL of a 140 mM K_2_S_2_O_8_ solution. One milliliter of the ABTS^•+^ was dissolved in 88 mL of PBS. In a quartz cell, the reaction contained 245 µL of an ABTS^•+^ and 5 µL of each test sample or dilutions of the Trolox standards. The absorbance was read within 80 min after the initial mixing. Calculations were made correlating the scavenging capacity of Trolox *versus* each sample. The antioxidant capacity was expressed as grams equivalent Trolox per mol of test sample.

### 3.4. Superoxide Dismutase-Like Activity

SOD activity was determined according to Beyer and Fridovich [16] with slight modifications as follows: the stock solution consisted of 2 mM xanthine (50 µL), 0.3% Triton X-100 solution (50 µL), 1.2 mM nitrobluetetrazolium (NBT, 50 µL), 0.1 mM phosphate buffer solution (770 µL, pH 7.8) and xanthine oxidase enzyme (600 mU mL^−1^, 20 µL) to start the reaction; 1 mM (60 µL) of each sample, **Fe_2_PO** or **Fe_2_PC** (for a final concentration of 60 µM) were added to the quartz cell by triplicate. Then, the quartz cell was mounted into a UV-visible spectrophotometer (Varian), and the absorbance was measured (at 540 nm for 5 min) as absorbance increment due to NBT formation per unit of time. Assays were performed at room temperature (25 °C). We reported the inhibition percentage of tetrazolium formazan formation, under the conditions describe above.

### 3.5. Peroxidase-Like Activity

POx activity was determined using the Amplex^®^ Red Hydrogen Peroxide/Peroxidase Assay Kit. The assay consisted in placing into individual wells of a microplate 50 µL of working solution prepared as follows: 10 mM Amplex^®^ Red (10-acetyl-3,7-dihydroxyphenoxazine) reagent stock solution (50 µL), 20 mM H_2_O_2_ solution (500 µL) and 1X Reaction Buffer (4.45 mL) and **Fe_2_PO** or **Fe_2_PC** (50 µL) were added with a final concentration of 0.1 mM. The POx activity was determined spectrophotometrically monitoring the oxidation of the Amplex^®^ Red reagent to Resourfin in the presence of H_2_O_2_ and a POx or POx mimetic, using a microplate reader at 550 nm after 0.5, 12, 24 and 48 h of incubation.

### 3.6. Cellular Assay

PBMCs were isolated from heparinized blood obtained from human healthy donors by a density gradient centrifugation using Ficoll-Hypaque. Isolated PBMCs (5 × 10^5^ cells per well) were resuspended in RPMI-1640 medium containing 10% heat-inactivated fetal calf serum, 50 mM 2-mercapoethanol, 100 U mL^−1^ amphotericin band seeded onto 96-well tissue culture plates for stimulation with phytohemagglutinin (PHA, 5 µg mL^−1^) in the presence of enzymatic system, xanthine plus xanthine oxidase (XO), as an enzymatic generator of reactive oxygen species, *i.e*., superoxide, hydrogen peroxide and hydroxyl radicals [16] (8 mM of xanthine and 23 mU mL^−1^ XO), and a different concentrations of **Fe_2_PO** and **Fe_2_PC** (0, 30, 60 and 120 µM). Cells were incubated 6, 18 and 24 h at 37 °C in 5% CO_2_. Cell viability was evaluated by trypan blue dye exclusion assay.

## 4. Conclusions

Two novel Fe(III)-cyclophanes have shown bioactive properties as antioxidants, reducing the synthetic ABTS^•+^ radical, within the range observed for ascorbic acid, which was used as the reference antioxidant. Reported results for similar Cu(II) complexes [5] demonstrated the biomimetic potential of these metallic cyclophane derivatives [7]. Aside of their antioxidant capacity comparable to phenolic acids, flavonoids and vitamins C and E [17] is the lack of need for negative interactions with cells. For that matter, a bioassay using PBMC cells injured with ROS was done to test their antioxidant capacity *in vivo*. In fact, both **Fe_2_PO** and **Fe_2_PC** were able to scavenge the superoxide produced by xanthine and xanthine oxidase, leading to an increased cellular survival.

The differences in biomimetic activity between **Fe_2_PO** and **Fe_2_PC** could be due to structural differences between the cyclophanes. The action of iron-cyclophanes against the superoxide radical was similar in the SOD assay, while when a stable radical (ABTS) was used to test antioxidant capacity, **Fe_2_PO** was better than **Fe_2_PC**. It is known that **PC** is more rigid compared to **PO**, and since ABTS is a large molecule with a mass over 500 Da, we postulate that steric effects arise at the interaction with the synthetic antioxidant. Considering that these molecules could remove superoxide radical and led to molecular water that not affect cells, they appear to be very interesting molecules for further studies to evaluate their antioxidant and bioactive properties in animal models and cancer cell lines.
